# HTLML: Hybrid AI Based Model for Detection of Alzheimer’s Disease

**DOI:** 10.3390/diagnostics12081833

**Published:** 2022-07-29

**Authors:** Sarang Sharma, Sheifali Gupta, Deepali Gupta, Ayman Altameem, Abdul Khader Jilani Saudagar, Ramesh Chandra Poonia, Soumya Ranjan Nayak

**Affiliations:** 1 Chitkara Institute of Engineering and Technology, Chitkara University, Punjab 140401, India; sarang.sharma@chitkara.edu.in (S.S.); sheifali.gupta@chitkara.edu.in (S.G.); deepali.gupta@chitkara.edu.in (D.G.); 2 Department of Computer Science and Engineering, College of Applied Studies and Community Services, King Saud University, Riyadh 11533, Saudi Arabia; aaltameem@ksu.edu.sa; 3 Information Systems Department, Imam Mohammad Ibn Saud Islamic University (IMSIU), Riyadh 11432, Saudi Arabia; 4 Department of Computer Science, CHRIST (Deemed to be University), Bangalore 560029, India; rameshchandra.poonia@christuniversity.in; 5 Amity School of Engineering and Technology, Amity University Uttar Pradesh, Noida 201301, India; nayak.soumya17@gmail.com

**Keywords:** Alzheimer’s disease, SVM, gaussian NB, XGBoost, DenseNet121, DenseNet201, deep learning, convolutional neural network

## Abstract

Alzheimer’s disease (AD) is a degenerative condition of the brain that affects the memory and reasoning abilities of patients. Memory is steadily wiped out by this condition, which gradually affects the brain’s ability to think, recall, and form intentions. In order to properly identify this disease, a variety of manual imaging modalities including CT, MRI, PET, etc. are being used. These methods, however, are time-consuming and troublesome in the context of early diagnostics. This is why deep learning models have been devised that are less time-intensive, require less high-tech hardware or human interaction, continue to improve in performance, and are useful for the prediction of AD, which can also be verified by experimental results obtained by doctors in medical institutions or health care facilities. In this paper, we propose a hybrid-based AI-based model that includes the combination of both transfer learning (TL) and permutation-based machine learning (ML) voting classifier in terms of two basic phases. In the first phase of implementation, it comprises two TL-based models: namely, DenseNet-121 and Densenet-201 for features extraction, whereas in the second phase of implementation, it carries out three different ML classifiers like SVM, Naïve base and XGBoost for classification purposes. The final classifier outcomes are evaluated by means of permutations of the voting mechanism. The proposed model achieved accuracy of 91.75%, specificity of 96.5%, and an F1-score of 90.25. The dataset used for training was obtained from Kaggle and contains 6200 photos, including 896 images classified as mildly demented, 64 images classified as moderately demented, 3200 images classified as non-demented, and 1966 images classified as extremely mildly demented. The results show that the suggested model outperforms current state-of-the-art models. These models could be used to generate therapeutically viable methods for detecting AD in MRI images based on these results for clinical prospective.

## 1. Introduction

AD is a neurological condition which damages the brain cells that slowly erodes memory and hampers basic cognitive functions and abilities. This disease is detected by changes in the brain and eventually results in neuron loss and their connections. According to WHO, around 50 million people have dementia, and there are nearly 10 million new cases of AD every year. Eventually the risk of developing AD reaches 50% for individuals beyond age 85. Ultimately, AD destroys the part of the brain that operates breathing and heart monitoring, eventually leading to fatality. AD consists of three stages: very mild, mild and moderate [1,2]. However, an individual affected with AD begins to show symptoms at a moderate stage, due to which communication between neurons becomes affected.

Progression of deterioration in the middle stage eventually impedes independence, with patients unable to execute many of the most routine daily tasks. Inability to recall vocabulary causes inappropriate word replacements, resulting in speech issues. The ability to read and write is also dwindling [3,4,5]. As Alzheimer’s disease develops, the risk of falling increases because complex motor sequences become less coordinated. In this stage, the person’s memory issues increase and he or she may not recognize close family members anymore [6,7]. Disruption in long-term memory causes problems. However, old age alone does not cause AD; several health, environmental and lifestyle factors also contribute to AD [8,9,10,11]. These include heart disease, lack of social engagement and lack of sleep, which also contribute to risk of developing AD. CT, MRI, and PET scans are among the tests that doctors use to diagnose this disease [12,13,14,15,16]. However, MRI pictures are widely relied upon by both doctors and radiologists [17,18,19]. Further, these MRI images are expensive as they require complex hardware and software.

A novel modified Inception utilized model has been proposed to classify AD in four classes, i.e., Very Mild Demented (V.M.D), Mild Demented (M.D), Moderate Demented (Mod.D) and Non Demented (N.D). Moreover, this model is executed and utilized for a bigger dataset. The accuracy has been updated by implementing pre-processing methods on the MRI images. The model ran on a large MRI dataset. The following contributions can be inferred from the paper as follows:

- A hybrid AI-based model was proposed by combining both transfer learning (TL) and permutation-based machine learning (ML) for AD diagnosis. The three hybrid DenseNet-121 models have been simulated with combinations of three machine learning classifiers, i.e., SVM, Gaussian naïve base and XGBoost, respectively, for detection of Alzheimer’s disease. From these models, the best hybrid DenseNet-121-SVM model was selected for further simulation.
- Two TL-based models were implemented, namely DenseNet-121 and DenseNet-201, for feature extraction.
- Finally, the three most popular machine learning (ML) classifiers, namely SVM, Gaussian naïve base and XGBoost, were respectively implemented for classification purposes.
- A permutation-based voting classifier was implemented for final accuracy observation.
- The proposed model was implemented using Adam optimizer and 1000 Epochs for evaluation purposes.

## 2. Background Literature

Most of the research work was implemented on the binary classification (Bin.c) of AD, and a smaller dataset was utilized to design their proposed model, which was not adaptable. Many of these authors have been training on a bigger dataset. They had performed binary classification. Table 1 compares previous models.

From Table 1, smaller datasets have been utilized to execute these models. However, the proposed model ran on a large dataset and does not utilize binary classification. Rather, it classifies Alzheimer’s disease into four categories, that is, MD, V.M.D, Mod. D and N.D.

## 3. Proposed Research Methodology

**Figure 1:**
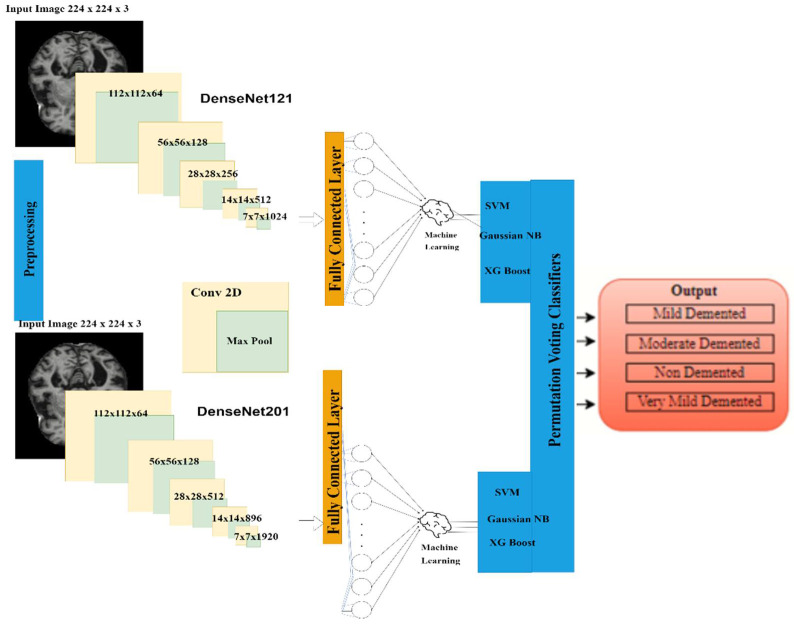
Block Diagram of Proposed Research Model.

A transfer learning-based DenseNet model is an ensemble with machine learning classifiers in which DenseNet is used to extract the feature map from the image dataset, whereas machine learning classifiers are used to classify the feature map into four categories, i.e., ND, VMD, MD and Mod D classes. For feature map extraction, two DenseNet models, i.e., Densenet121 and DenseNet201 are used. For classification, three types of classifiers are used, i.e., SVM, Gaussian NB and XG Boost. The proposed model utilizes the Kaggle dataset containing 6200 AD images. The framework consists of various layers as shown in Figure 1. The different blocks of the proposed model are explained below. The model is executed using the Keras package in Python with Tensorflow, which is used at the backend on an Intel(R) Core(TM) i5-6400 CPU 2.70 GHz processor and 12GB RAM.

### 3.1. Input Dataset

**Figure 2:**
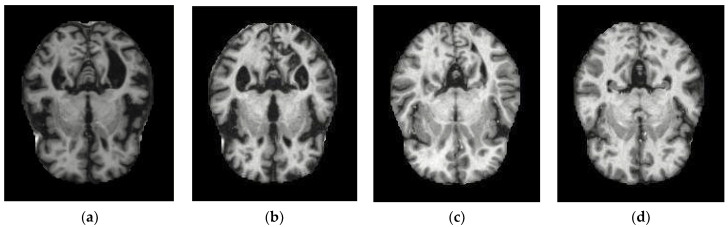
Alzheimer’s Disease MRI Dataset: (**a**) M.D, (**b**) Mod.D (**c**) N.D and (**d**) V.M.D.

The database used in the study consists of a total of 6126 AD images that were collected from the Kaggle database. It comprises grayscale images of 896 MD, 64 Mod D, 3200 ND and 1966 VMD of dimensions (208 × 176 × 3) pixels. The dataset for evaluation was divided in such a way that 80% of the data were utilized for training and the remaining 20% for testing. Table 2 shows the number of images used for training and validation purposes. Figure 2 shows the database sample images for each category i.e., M.D, V.M.D, Mod. D and N.D.

### 3.2. Data Pre-Processing

Data preprocessing is a technique of assembling input data and making them utilizable for deep learning models [20,21,22]. It is the fundamental step in this proposed research methodology. Data preprocessing is required for cleaning the noise, identifying and rectifying the missing values, and making the data usable in an appropriate format [23,24]. Further, this enhances the efficacy of the whole proposed research model [25,26]. In this model, two types of data pre-processing are utilized, namely data normalization and data augmentation.

#### 3.2.1. Data Normalization

Data normalization preserves the numerical stability of the modified Inception model. MRI images were acquired basically in grayscale format. Utilizing normalization techniques, the MRI datasets in the proposed model were trained faster.

#### 3.2.2. Data Augmentation

**Figure 3:**
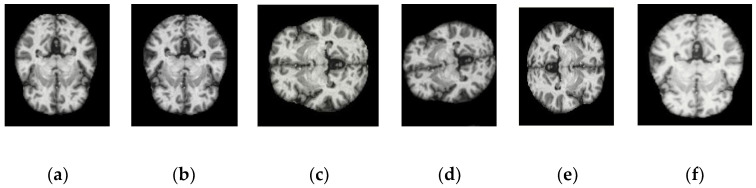
Flipping applied to dataset (**a**) original (**b**) horizontal flipping (**c**) vertical flipping (**d**) 90 degree anticlockwise (**e**) 270 degree anticlockwise and (**f**) brightness factor 0.7.

In order to enhance the usefulness of the model, a large dataset is needed. However, numerous sites as well as privacy and data restrictions are issues faced while acquiring these datasets. In order to overcome these problems, various augmentation methods on the dataset were implemented. These augmentation methods significantly expanded the original data quantity. Techniques such as Horizontal Flipping, Vertical Flipping, and Anticlockwise Rotated Image at 900, Rotated Image at 2700 and Brighter Image by a factor 0.7 are implemented. These five data augmentation methods are shown in Figure 3.

Table 3 exhibits the quantity of images before and after data augmentation. Further, there is a disproportion in the number of images in every class. In order to improve this disproportion, the above processes were performed. After performing these methods, the original dataset was updated to 10,760 images. Table 3 represents the number of newly updated images. The augmentation was applied only on the training images. Earlier, the training images of MD, Mod D, ND and VMD were 896, 64, 3200 and 1966, respectively. After the augmentation, training images totaled 10,760 in count. Table 3 also shows the total images of training and validation data after augmentation.

### 3.3. Feature Extraction Using Different DenseNet Transfer Learning Models

In the proposed model, input images with 208 * 176 sizes are applied to two DenseNet models, i.e., DenseNet121 and DenseNet201, for feature map extraction. The DesneNet121 model consists of five convolutional blocks as shown in Table 4. In the first convolution block (Block-1), the image is shaped to match the Conv_1 size 112 × 112, then it is transferred to the max pooling block. From the max pooling block, it is then sent to Conv_2 to again to be shaped into 56 × 56, then to Conv_3 for 28 × 28, Conv_4 for 14 × 14, and finally to Conv_5 for 7 × 7. After Conv_5, the obtained features are then sent to the global average pooling layer and finally sent to the dense layer to be obtained as output. Similarly, layers description of DenseNet201 is shown in Table 5. The difference between DenseNet121 and DenseNet201 is in the number of convolution layers used in each convolution block. The last dense layer is also different in both DenseNet structures; 1024 filters are used in DenseNet121, whereas DenseNet201 uses 1920 filters.

#### 3.3.1. Feature Extraction Using DenseNet 121 Model

Table 6 exhibits both the filter visualization images of every convolution layer of DenseNet 121 after every dense block. There are a total of five convolutional blocks in the proposed model. The single kernel or filters along with the images after every convolution layer are shown in Table 6.

#### 3.3.2. Feature Extraction Using DenseNet 201 Model

Table 7 exhibits both the filter visualization image of every convolution layer of DenseNet 201 as well as filtered images of each class after every dense block. There are a total of five convolutional blocks in the proposed model. The single kernel or filters along with the images after every convolution layer are shown in Table 7.

### 3.4. Classification Using Hybrid Machine Learning-Convolutional Neural Network

The extracted features obtained from Block-5 of both DenseNet architectures are sent to Machine Learning Classifiers, namely SVM, XG Boost and Gaussian NB. After that, these are finally sent to a dense layer to be obtained as output. The layer description of both hybrid architectures is shown in Table 8 and Table 9, respectively, in which there are two major differences—one is in the number of filters used at each layer and another is the number of times a block runs.

#### 3.4.1. Gaussian Naïve Bayes Classifier

These are supervised machine learning classification techniques which are based on Bayes’ theorem. They can be used to calculate conditional probability. The value of individual features in these classifiers is totally independent. These features do not depend on the values of any other features. This classifier utilizes continuous data that usually take continuous values associated with their respective class. The likelihood feature is given by Equation (1).

(1) $$P(x=v|C_{k})=\frac{1}{\sqrt{2\pi \sigma^{2}{}_{k}}}exp\left(-\frac{{\left(v-{µ}_{k}\right)}^{2}}{2\sigma^{2}{}_{k}}\right)$$

where ${µ}_{k}$ is mean of values in ***x*** associated with class $C_{k}$, and $\sigma^{2}{}_{k}$ is Bessel corrected variance; ***v*** is a random observation value.

#### 3.4.2. XGBoost Classifier

XG Boost stands for Extreme Gradient Boosting, which is a fully optimized distributed gradient boosting module which tends to be highly flexible, portable and efficient. For unstructured datasets like image datasets, this classifier is highly utilized along with several convolutional neural network models. This classifier is mainly utilized for higher unstructured datasets. XG Boost is given in Equation (2).

(2) $$f\left(x\right)=f_{M}\left(x\right)=\sum_{m=0}^{M}((f_{m}\left(x\right)=\sum_{m=0}^{M}))\left(f_{\left(m-1\right)}\left(x\right)+f_{\left(m\right)}\left(x\right)\right)$$

where *x* is an input training set trained by weak learners ranging from *m* to *M*, $f_{\left(m\right)}\left(x\right)$ defines model update.

#### 3.4.3. Support Vector Machine Classifier

This technique is utilized for each data item as n-dimensional space with each feature value as coordinate value. Then, the hyper-plane is obtained by distinguishing the specified classes. SVM classifier is given by Equation (3).

(3) $$f\left(x\right)=\left[\frac{1}{n}\sum_{i=1}^{n}(max\left(0,1-y_{i}\left(w^{T}x_{i}-b\right))\right)\right]+\lambda {\left|\left|w\right|\right|}^{2}$$

where $x_{i}$ is *i*th dimensional real vector, $y_{i}$ indicates the *i*th class to which $x_{i}$ belongs, ***w*** is normal vector to hyperplane, $w^{T}x_{i}-b$ gives the *i*th output, *n* gives total number of input points, and λ is a hard margin classifier for classifiable input data.

## 4. Results Analysis

The hyper parameters are utilized and essential for tuning the model, which may include optimizer, batch size (BS) and epochs. These optimization techniques are used to reduce the losses that have already occurred. Optimizers are algorithms or methods used to modify neural network features so as to minimize the losses. Adam optimizer was used in this model. BS specifies managed images in a single iteration. BS 64 was utilized in these models. Epochs indicate the number of times the dataset has been received by the neural network. One thousand epochs were used in these models. The Adam optimizer is used for training the deep learning algorithms, as it combines both AdaGrad and RMSProp optimizers’ characteristics. A large BS causes heavy computational processes during deep learning model training. However, small BS allows faster computational processes. Hence, there is always a trade-off between large and small BS. The number of epochs should be more so that error can be minimized during model training; however, a large number of epochs increases the computational time. Hence, there should be a trade-off between a high and small number of epochs. Table 8 shows the name of hyper tuning parameters and their values.

### 4.1. Analysis of Hybrid DenseNet 121 Model

The features extracted from the Densenet121 model are classified using three different machine learning classifiers, namely SVM, Gaussian NB and XG Boost. The performance of these three hybrid DenseNet121 models is analyzed using train and validation loss and confusion matrix parameters.

#### 4.1.1. Training and Validation Loss of Hybrid DenseNet121 Models with Different Epochs

**Figure 4:**
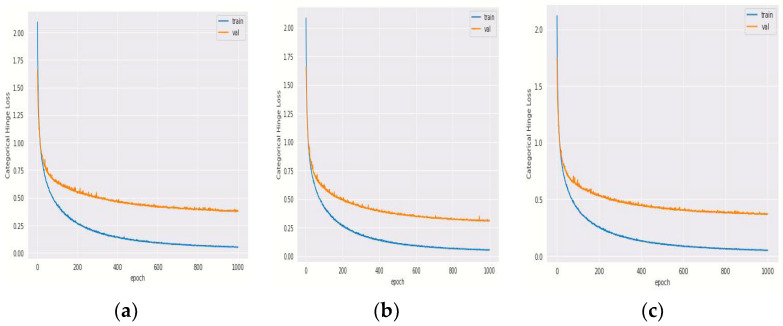
Categorical Hinge Loss vs. Epoch Curve for hybrid DenseNet121 model with classifiers (**a**) SVM, (**b**) Gaussian NB and (**c**) XG Boost.

Hinge loss, which depicts changes in loss during model training, is shown in Figure 4, and minimum hinge loss is achieved for hybrid DenseNet121-SVM (Figure 4a) and hybrid Desnenet121-Gaussian NB (Figure 4b), whereas validation hinge loss is more for hybrid DenseNet121-XG Boost (Figure 4c).

The performance parameters of hybrid DenseNet 121 models for different epochs are depicted in Table 10, in which hybrid DenseNet121-SVM outperforms at 1000th epoch. At 1000th epoch, the training loss is 0.051, and validation loss is at 0.313 for DenseNet121 with SVM.

#### 4.1.2. Confusion Matrix Comparison for Hybrid DenseNet121 Models

**Figure 5:**
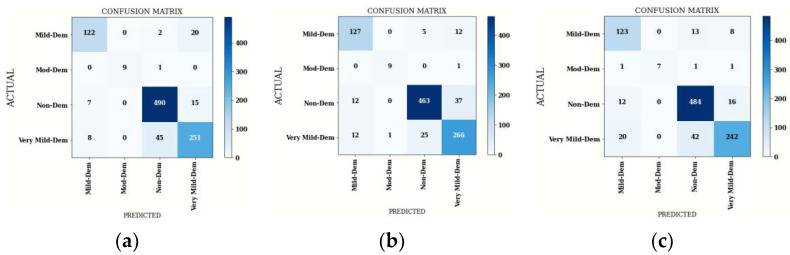
Confusion Matrix of Hybrid DenseNet121 with Three Machine Learning Classifiers: (**a**) SVM, (**b**) Gaussian NB and (**c**) XG Boost.

**Figure 6:**
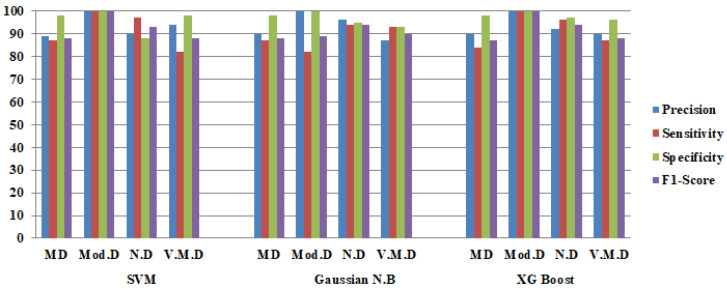
Confusion Matrix Parameters of Hybrid DenseNet121 with Three Machine Learning Classifiers.

The confusion matrices of the hybridDenseNet121 models with machine learning classifiers are shown in Figure 5. Both true and false predictions are displayed by these matrices. Each column is identified by its class name, viz. M.D, Mod.D, N.D and V.M.D. The precise number of images classified by a particular model can be determined using diagonal values. The accuracy of all DL models is shown in Figure 6, where DenseNet121 with SVM performs better than other DL Models with machine learning classifiers.

From Table 11, the average performance comparison of all the DenseNet121 hybrid models with average Precision(P), average Sensitivity(S), average Specificity (Sp), average F1-Score (F1) and average accuracy is depicted. These average parameters are obtained by using batch size 64. It can be seen that a stable and better testing performance is achieved with DenseNet121-SVM.

### 4.2. Analysis of Hybrid DenseNet 201 Model

The features extracted from the DenseNet201 model are classified using three different machine learning classifiers, namely SVM, Gaussian NB and XG Boost. The performance of these three hybrid DenseNet201 models is analyzed using training and validation loss and confusion matrix parameters.

#### 4.2.1. Training and Validation Loss of Hybrid DenseNet201 Models with Different Epochs

**Figure 7:**
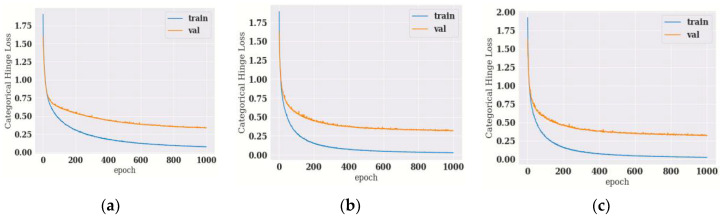
Categorical hinge loss vs. epoch curve for hybrid DenseNet201 model with classifiers (**a**) SVM, (**b**) Gaussian NB and (**c**) XG Boost.

Hinge loss, which depicts changes in loss during model training, is shown in Figure 4. Minimum hinge Loss is achieved for DenseNet201-SVM (Figure 7a) and DenseNet201-Gaussian NB (Figure 7b), whereas validation hinge loss for DenseNet201-XGBoost (Figure 7a) is more than that of the other two hybrid models.

The performance parameters of hybrid DenseNet 201 models for different epochs are depicted in Table 12, in which hybrid DenseNet201-Gaussian NB outperforms at 1000th epoch. The hybrid DenseNet201-Gaussian NB outperforms the remaining three DL models at 1000th epoch. The minimum training loss is 0.028, and validation loss is at 0.265 for DenseNet 201 with Gaussian NB.

#### 4.2.2. Confusion Matrix Comparison for Hybrid DenseNet201 Model

**Figure 8:**
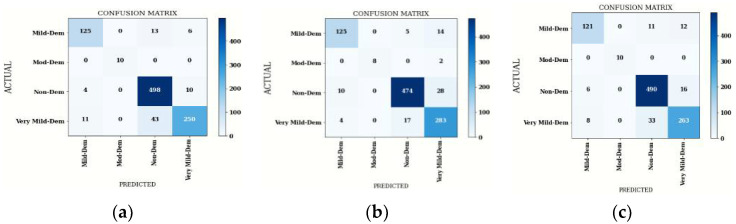
Confusion Matrix of DenseNet201 with Three Machine Learning Classifiers: (**a**) SVM, (**b**) Gaussian NB and (**c**) XG Boost.

**Figure 9:**
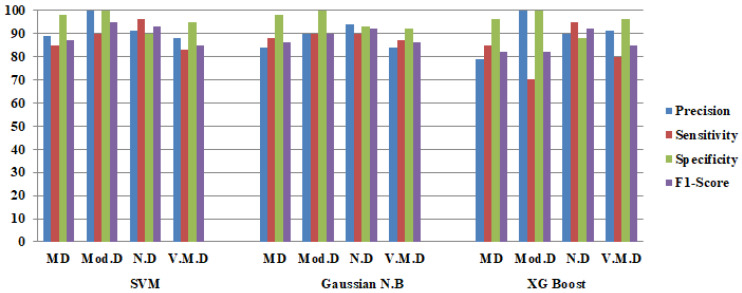
Confusion Matrix Parameters of DenseNet201 with Three Machine Learning Classifiers.

The confusion matrices of the DenseNet201 model with machine learning classifiers of batch size 64 are shown in Figure 8. Both true and false predictions are displayed in these matrices. The accuracy of all DL models is shown in Figure 9, where DenseNet201 with Gaussian NB performs better than the other DL Models with machine learning classifiers.

From Table 13, the average performance comparison of all DenseNet201 hybrid models with average Precision, average Sensitivity, average Specificity, average F1-Score and average accuracy is depicted. These average parameters are obtained by using batch size 64. It can be seen that a stable and better testing performance is achieved with DenseNet201-Gaussian NB.

### 4.3. Comparison of Hybrid DenseNet121-SVM and DenseNet201-GNB Classifier

**Figure 10:**
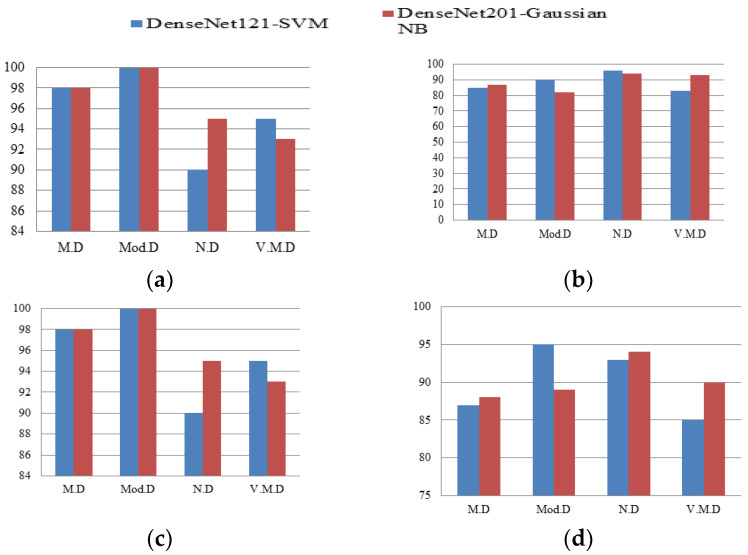
Confusion Matrix Parameters of both Hybrid DenseNet Models with Machine Learning Classifiers depicting (**a**) Precision, (**b**) Sensitivity, (**c**) Specificity and (**d**) F1-Score.

Precision, sensitivity, specificity and F1-Score of both DenseNet121-SVM and DenseNet201-Gaussian NB models are depicted from Figure 10a–d, respectively. From Figure 10a, DenseNet201-Gaussian NB performs better for both M.D and N.D, whereas DenseNet121-SVM performs better for V.M.D. Both models perform best for Mod.D. From Figure 10b, DenseNet201-Gaussian NB performs better for both M.D and V.M.D, whereas DenseNet121-SVM performs better for both Mod.D and N.D. From Figure 10c, DenseNet201-Gaussian NB performs better for N.D, whereas DenseNet121-SVM performs better for V.M.D. Both models perform better for both M.D and Mod.D. From Figure 10d, DenseNet201-Gaussian NB performs better for M.D, N.D and V.M.D, whereas DenseNet121-SVM performs better only for Mod.D.

**Figure 11:**
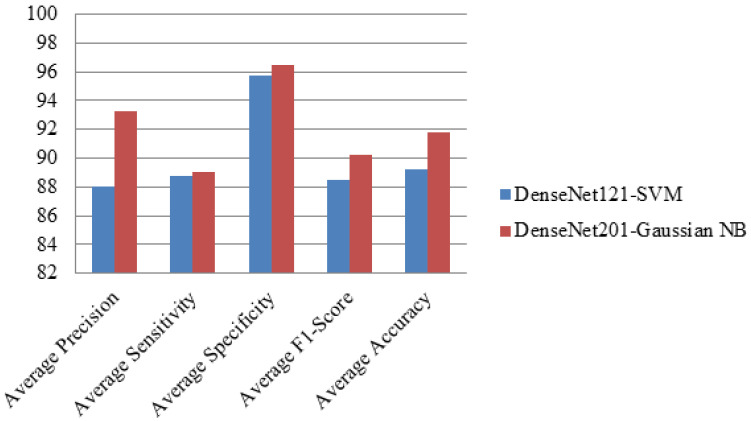
Average Performance Parameters of both Hybrid DenseNet Models with Machine Learning Classifiers.

Average precision, average sensitivity, average specificity, average F1-score and average accuracy of both DenseNet121-SVM and DenseNet201-Gaussian NB models are depicted in Figure 11. From Figure 11, DenseNet201-GaussianNB outperforms DenseNet121-SVM in all these criteria, such as average precision, average sensitivity, average specificity, average F1-score and average accuracy.

### 4.4. State of Art Comparison

Results obtained from pre-trained D.L models are shown in comparison with previous models using MRI images as shown in Table 14. The utilized approach outperformed other previous approaches. This approach utilized DenseNet121 and DenseNet201 with preprocessing and machine learning classifier methods to modify their efficacy.

## 5. Conclusions

This paper displayed the usefulness of DL models for prediction of AD. DenseNet201 outperforms DenseNet121 in various comparative parameters. The dataset was acquired from Sarvesh Dubey via Kaggle. Accuracy of 91.75%, specificity of 96.5% and F1-score of 90.25%, respectively, were achieved with the DenseNet201 for Gaussian NB. These results would help radiologists to obtain a second opinion or simulator.

The model performs better when both environments for training and testing are similar. A possible limitation would be to guarantee reproducibility; however, the issue could be resolved through collections of large brain MRI datasets. A hybrid approach places the convolutional information into machine learning parts and the AD images into deep learning parts before adding the results of both processes. Medical imaging requires various DL techniques for various bio-medical applications. Further, progress will be made on this model to overcome issues with modifying image acquisition, enhancement, different data formats integration, and weights misalignment, while applying the model to specific AD problems. As more data are acquired, the research could be made more impactful. Further, expanding from 2DCNN to 3DCNN could also be achieved, which mostly deals with multimodal aspects of brain MRI images. For data augmentation, GAN could also be implemented. Reinforcement learning, which makes its own decisions based on the existing environment, could also be used. This approach is still evolving to achieve better performance and transparency. As AD image data and computer assets are growing rapidly, research on AD using deep learning involving hybrid methods is also continuously evolving. This would be a necessary boon not only to these applications but to new approaches presently conducted in medical institutions as well.

## Figures and Tables

**Table 1:** Comparison of existing state-of-art models.

Citation	Approach	Objective	Challenges of the Approach
[1]	Non Linear SVM with 2D CNN	To develop an automated technique to classify normal, early and late mild AD subjects.	Dataset consisted of 1167 MRI images. It was able to achieve 75% while performing Bin.c.
[2]	2D CNN, 3D CNN 3D CNN-SVM	To distinguish AD and MCI individuals from normal individuals and to improve value based care of affected individuals in medical facilities.	Dataset contained 3127, 3T T1-weighted images. It performed tertiary classification and was able to an accuracy of 88.9%. It also aims to focus on reverting MCI individuals to normal individuals, predict AD progression and improve diagnosis of AD in future.
[3]	GoogleNet, AlexNet, VGGNet16VGGNet19SqueezeNetResNet18 ResNet50 ResNet101Inceptionv3.	To detect AD on MRI scans using D.L techniques.	Dataset consisted of 177 images. It performed Bin.c and achieved an accuracy of 84.38%. To include other neuro-imaging modalities such as PET scans or features in the system to take different aspects of AD into consideration.
[4]	Data Augmentation, CNN.	To classify AD by using Cross-Modal Transfer Learning	Dataset contained 416; sMRI image scans and it implemented Bin.c and achieved an accuracy of 83.57%. To proceed with a longitudinal dataset and develop a method based on spatial optimization of ROI.
[5]	DTCWT, PCA, FNN	To develop a CAD system to early diagnose AD individuals.	Dataset contained 416; T1- weighted image scans and it performed Bin.c and achieved an accuracy of 90.06%. Various feature reduction methods such as ICA, LDA and PCA were utilized for swarm optimization.
[6]	SVM, CNN	To classify AD from MCI by using semi-supervised SVM-CNN.	Dataset contained 359; T1- weighted images and it performed Bin.c and achieved an accuracy of 82.91%. To distinguish brain MRI images semi semi-supervised SVM is applied.
[7]	SVM-REF, CNN	To classify AD by using SVM-REF-CNN.	Dataset contained 1167; T1-weighted image scans and it performed Bin.c and achieved an accuracy of 81%. To distinguish brain images by using SVM-REF.
[8]	2D-CNN, VGG16	To classify AD by using ensemble based CNN.	Dataset contained 798; T1-weighted image scans and it performed Bin.c and achieved an accuracy of 90.36%. To distinguish AD from MCI images by using 2D-CNN.
[9]	SVM, CNN	To distinguish MCI from AD by using an SVM classifier with a linear kernel.	Dataset contained 1167; T1-weighted image scans and it performed Bin.c and achieved an accuracy of 69.37%. To distinguish AD from MCI images by using SVM-CNN.
[10]	SVM, k-NN, CNN	To distinguish MCI from AD by using SVM and k-NN.	Dataset contained 1311; T1 & T2 weighted image scans and it performed Bin.c and achieved an accuracy of 75%. To distinguish AD from MCI images by using SVN-CNN, KNN.

**Table 2:** Kaggle available Alzheimer’s Disease Dataset.

Dataset Source	Class Name	Training Images	Validating Images	Total Images
Kaggle	M.D Mod.D V.M.D N.D	717 52 2560 1518	179 12 640 448	896 64 3200 1966

**Table 3:** Alzheimer’s Dataset with Augmentation.

S.No.	Name of the Class	Number of Images before Augmentation	Images after Augmentation	
			Training Images	Validating Images
1	M.D	896	2150	538
2	Mod.D	64	512	128
3	N.D	3200	2800	700
4	V.M.D	1966	3145	787

**Table 4:** Layers Description of Conventional Neural Network DenseNet121.

Block Name	Layer Name	Input Size	Output Size	Filter Size	Number of Filters	Number of Times Block Run
Conv_1	Conv_1_1	224 × 224	112 × 112	7 × 7	64	1
Conv_2	Conv_2_1:Conv_2_6	112 × 112	56 × 56	1 × 1	128	6
Conv_3	Conv_3_1:Conv_3_12	56 × 56	28 × 28	1 × 1	256	12
Conv_4	Conv_4_1:Conv_4_48	28 × 28	14 × 14	1 × 1	512	48
Conv_5	Conv_5_1:Conv_5_32	14 × 14	7 × 7	1 × 1	1024	32

**Table 5:** Layers Description of Conventional Neural Network 201.

Block Name	Layer Name	Input Size	Output Size	Filter Size	Number of Filters	Number of Times Block Run
Conv_1	Conv_1_1	224 × 224	112 × 112	7 × 7	64	1
Conv_2	Conv_2_1:Conv_2_12	112 × 112	56 × 56	1 × 1	128	12
Conv_3	Conv_3_1:Conv_3_24	56 × 56	28 × 28	1 × 1	512	24
Conv_4	Conv_4_1:Conv_4_96	28 × 28	14 × 14	1 × 1	896	96
Conv_5	Conv_5_1:Conv_5_64	14 × 14	7 × 7	1 × 1	1920	64

**Table 6:** Filter visualization and image conception for convolution layers of DenseNet121.

Name of Corresponding Block	Filter for First Convolution Layer	Image for First Convolution Layer	Filter for Last Convolution Layer	Image for Last Convolution Layer
Conv_1	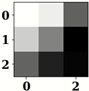	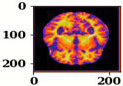	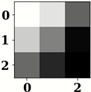	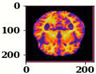
Conv_2	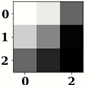	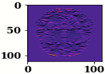	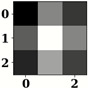	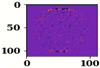
Conv_3	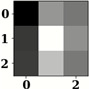	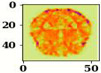	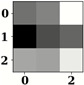	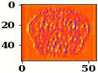
Conv_4	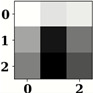	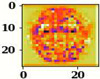	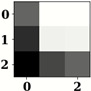	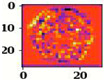
Conv_5	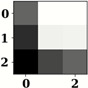	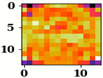	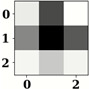	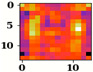

**Table 7:** Filter visualization and image visualization for each convolution layer of DenseNet 201.

Name of Block	Filter for First Convolution Layer	Image for First Convolution Layer	Filter for last Convolution Layer	Image for Last Convolution Layer
Conv_1	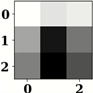	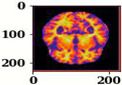	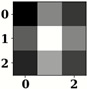	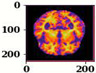
Conv_2	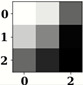	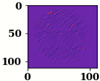	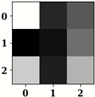	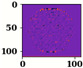
Conv_3	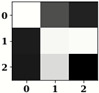	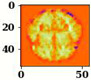	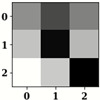	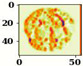
Conv_4	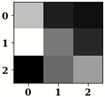	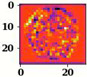	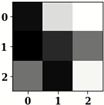	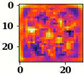
Conv_5	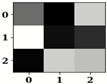	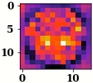	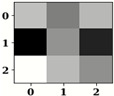	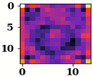

**Table 8:** Layers Description of Hybrid DenseNet121 Model.

Block Name	Layer Name	Input Size	Output Size	Filter Size	Number of Filters	Number of Times Block Run
Conv_5	Conv_5_1:Conv_5_32	14 × 14	7 × 7	1 × 1	1024	32
Conv_5	Machine Learning Classifiers	7 × 7	4 × 1	1 × 1	1024	32
Dense_4	Dense	4 × 1	4 × 1	N.A	N.A	1

**Table 9:** Layers Description of Hybrid DenseNet201 Model.

Block Name	Layer Name	Input Size	Output Size	Filter Size	Number of Filters	Number of Times Block Run
Conv_5	Conv_5_1:Conv_5_64	14 × 14	7 × 7	1 × 1	1920	64
Conv_5	Machine Learning Classifiers	7 × 7	4 × 1	1 × 1	1920	64
Dense_4	Dense	4 × 1	4 × 1	N.A	N.A	1

**Table 10:** Training and Validation Loss of Hybrid DenseNet121 Model with Varying Epochs and Fixed Batch Size 64.

SVM			GNB		XG	
Epoch	Train Loss	Valid Loss	Train Loss	Valid Loss	Train Loss	Valid Loss
200	0.264	0.554	0.265	0.497	0.262	0.531
400	0.14	0.467	0.141	0.402	0.125	0.45
600	0.089	0.422	0.088	0.348	0.086	0.405
800	0.068	0.394	0.065	0.323	0.059	0.384
1000	0.051	0.313	0.051	0.38	0.05	0.372

**Table 11:** Confusion Matrix Parameters of Hybrid DenseNet121 Model (in %).

SVM					GNB				XG			
Type	P	S	Sp	F1	P	S	Sp	F1	P	S	Sp	F1
Average	92	89	96	90	88	89	96	89	90	83	95	85
Accuracy	89.89				89.18				88.25			

**Table 12:** Training and Validation Loss of Hybrid DenseNet201 Model with Varying Epochs and Fixed Batch Size 64.

SVM			GNB		XG	
Epoch	Train Loss	Valid Loss	Train Loss	Valid Loss	Train Loss	Valid Loss
200	0.16	0.418	0.158	0.427	0.157	0.459
400	0.075	0.326	0.073	0.348	0.07	0.373
600	0.047	0.294	0.047	0.317	0.046	0.353
800	0.035	0.292	0.033	0.299	0.031	0.326
1000	0.027	0.291	0.028	0.265	0.025	0.318

**Table 13:** Confusion Matrix Parameters of DenseNet201 (in %).

SVM					GNB				XG			
Type	P	S	Sp	F	P	S	Sp	F	P	S	Sp	F
Average	93	92	96	92	93	89	97	90	93	92	98	92
Accuracy	91.03				91.75				91.13			

**Table 14:** Comparison with existing state-of-art models.

Study	Dataset Source	No. of Images	Technique Used	Accuracy
Rallabandi et al. [1]	ADNI	1167	SVM with D.L	75%
Feng et al. [2]	ADNI	3127	2D-CNN with D.L	82.57%
Ebrahimi-Ghahnavieh et al. [3]	ADNI	177	DenseNet-201 ResNet50	84.38% 81.25%
Aderghal, K. et al. [4]	OASIS	416	Cross-Modal Transfer Learning	83.57%
Jha et al. [5]	OASIS	416	DTCWT and PCA with FNN	90.06%
Filipovych et al. [6]	ADNI	359	SVM, CNN	82.91%
Rathore et al. [7]	ADNI	1167	SVM-REF, CNN	81%
Kang et al. [8]	ADNI	798	2D-CNN, VGG16	90.36%
Li et al. [9]	ADNI	1167	SVM, CNN	69.37%
Venugopalan et al. [10]	ADNI	1311	SVM, k-NN, CNN	75%
Proposed Methodology	Kaggle	6400	DenseNet201-Gaussian NB	91.75%
			DenseNet201-XG Boost	91.13%
			DenseNet201-SVM	91.03%

## Data Availability

The data presented in this study are available on request from the corresponding author.
